# Clinical characteristics of attention-deficit/hyperactivity disorder in children and adolescents: association with quality of life and behavioral aspects

**DOI:** 10.1590/1984-0462/2022/40/2020342

**Published:** 2021-10-04

**Authors:** Marcone de Souza Oliveira, Mayke Felipe Dias Marinho, Stela Maris Aguiar Lemos

**Affiliations:** a Universidade Federal de Minas Gerais, Belo Horizonte, MG, Brazil.; b Faculdade Única, Ipatinga, MG, Brazil.

**Keywords:** Attention deficit disorder with hyperactivity, Quality of life, Comorbidity, Transtorno do déficit de atenção com hiperatividade, Qualidade de vida, Comorbidade

## Abstract

**Objective:**

To identify associations between clinical characteristics of children with attention-deficit/hyperactivity disorder (ADHD) and their sociodemographic aspects, quality of life, and results from the strengths and difficulties questionnaire.

**Methods:**

This is an observational analytical cross-sectional study with a non-probabilistic sample consisting of 72 children diagnosed with ADHD, aged 6 to 13 years, treated at 2 neuropediatric outpatient clinics. The instruments used were the Multimodal Treatment Study of Children with Attention-Deficit/Hyperactivity Disorder – Swanson, Nolan, and Pelham, version IV (MTA-SNAP-IV), the Strengths and Difficulties Questionnaire (SDQ), the Brazilian Economic Classification Criteria (CCEB), and the Quality of Life Assessment Scale for Children and Adolescents (AUQEI). We performed descriptive, bivariate, and multivariate analyses, considering a 5% significance level.

**Results:**

SDQ results were associated with abnormal MTA-SNAP-IV results (inattentive/hyperactive/combined). A 1-point increment in the SDQ score increased by 36.5% the likelihood of the child having an abnormal MTA-SNAP-IV classification. Regarding AUQEI, 30.6% of participants perceived their quality of life as poor and 69.4% as good.

**Conclusions:**

A higher SDQ score increased the child's chance of having an abnormal MTA-SNAP-IV result.

## Resumo

**Objetivo:**

Verificar a associação entre características clínicas de crianças com transtorno do déficit de atenção e hiperatividade (TDAH) e aspectos sociodemográficos, qualidade de vida e questionário de capacidades e dificuldades.

**Métodos:**

Estudo observacional, analítico e transversal, com amostra não probabilística composta de 72 crianças diagnosticadas com TDAH, com idade entre 6 e 13 anos, atendidas em dois ambulatórios de neuropediatria. Os instrumentos utilizados foram o Multimodal Treatment Study Swanson, Nolan e Pelham (MTA-SNAP-IV), de triagem para sinais e sintomas de pessoas com TDAH, o Questionário de Capacidades e Dificuldades (SDQ), o Critério de Classificação Econômica Brasil (CCEB) e a Escala de Avaliação de Qualidade de Vida em Crianças e Adolescentes (AUQUEI). Foram realizadas análises descritiva, bivariada e multivariada, considerando-se o nível de significância de 5%.

**Resultados:**

Os resultados do SDQ tiveram associação com o resultado alterado do MTA-SNAP-IV (desatento/hiperativo/misto). O aumento de 1 ponto no escore do SDQ elevou em 36,5% a chance de a criança ser classificada como alterada no MTA-SNAP-IV. Em relação ao AUQUEI, 30,6% dos participantes avaliaram a qualidade de vida como ruim e 69,4% como boa.

**Conclusões:**

O aumento no escore do SDQ elevou a chance de a criança ter o MTA-SNAP-IV alterado.

## INTRODUCTION

Attention-deficit/hyperactivity disorder (ADHD) is a neurobiological disorder that can affect people from childhood to adulthood.^1^ It results from the heterogeneous and complex interaction between genetic and environmental factors.^2^

The main ADHD symptoms are inattention, hyperactivity, and impulsivity, occurring in different situations. According to the Diagnostic and Statistical Manual of Mental Disorders (DSM-5),^3^ ADHD has three subtypes: predominantly hyperactive-impulsive (ADHD-HI), inattentive (ADHD-I), and combined (ADHD-C), with both the hyperactive and inattentive types.

The worldwide prevalence of children with ADHD is around 5%.^4^ Two-thirds of this population presents comorbidities, particularly externalizing behavior problems, such as oppositional defiant disorder (ODD) and conduct disorder (CT), with up to 40%, in addition to internalizing problems, including anxiety, depression, and obsessive-compulsive disorders, which affect up to 50% of these cases.^5^

A study carried out in Riyadh, Saudi Arabia, showed that ADHD in childhood is associated with impairment in academic performance, family interaction, peer relationships, and self-esteem, as well as a worsening in quality of life (QoL).^6^

Thus, this study aimed to identify associations between clinical characteristics of children with ADHD and their sociodemographic aspects, QoL, and results from the strengths and difficulties questionnaire.

## METHOD

This is an observational analytical cross-sectional study with a non-probabilistic sample consisting of children treated at two neuropediatric outpatient clinics in the Vale do Aço region, Minas Gerais, Brazil — one exclusive for the public health system (*Sistema Único de Saúde* — SUS) and the other for individuals who have private health insurance.

The sample size was estimated considering prevalence studies and associations between the outcome and independent variables. For the calculation, we adopted a 9% sampling error, 95% confidence interval (95%CI), and 50% prevalence, taking into account the range of the outcome of interest.

The inclusion criteria were: individuals aged 6 to 13 years; having a clinical diagnosis made by a neuropediatrician and receiving follow-up for ADHD; informed consent form (ICT) signed by the guardian; and assent form (AF) signed by the child. The exclusion criteria were: children who were not clinically evaluated; whose guardian did not completely answer the instruments; those living in shelters; and the ones who presented history or evidence of severe abnormalities in clinical data, such as neuromotor dysfunction, intellectual disability, and cognitive, neuromotor, or other psychiatric changes (e.g., schizophrenia and drug addiction). The exclusion of the disorders mentioned above followed the DSM-5 criteria.

Data were collected at neuropediatric outpatient clinics, where the guardians of children with ADHD (according to DSM-5 diagnostic criteria) were approached and informed about the study by reading the ICF and AF.

Children whose guardians agreed to participate in the investigation were submitted to a standard evaluation with completion of a specific medical protocol and subsequently referred to an office in the same clinic, where a single qualified Psychology intern, who at the beginning of the research was attending the seventh undergraduate semester, administered the questionnaire and collected the signatures of the forms. The intern was properly trained for this work. After administration of the instruments, the attending physician supervised the case.

The guardians answered the following instruments:

- Strengths and Difficulties Questionnaire (SDQ),^7^ which screens for child mental health problems and was validated in Brazil in 2000.^8^ This questionnaire investigates social behavior and problems related to child and adolescent mental health. SDQ consists of 25 semi-structured questions, divided into the following classes: prosocial behavior, hyperactivity, and emotional, conduct, and peer problems. The response categories are: “not true,” scored 0 or 2; “somewhat true,” scored 1; and “certainly true,” scored 0 or 2. After data collection, all items were added up. A total score greater than or equal to 20 was defined as abnormal (probable psychiatric disorder), between 16 and 19 as borderline, and lower than or equal to 15 as normal;
- Multimodal Treatment Study of Children with Attention-Deficit/Hyperactivity Disorder – Swanson, Nolan, and Pelham, version IV (MTA-SNAP-IV),^9^ which includes 26 items related to signs and symptoms of inattention, hyperactivity, and the combination of both described in DSM-5 for ADHD and ODD. Questions 1 to 9 correspond to inattention; 10 to 18 to hyperactivity/impulsivity; and 19 to 26 to oppositional/defiant characteristics. Each answer adopted the following score: 0 for “not at all”, 1 for “just a little”, 2 for “quite a bit”, and 3 for “very much”. The score was calculated by adding the points up and dividing the result by 26;
- Brazilian Economic Classification Criteria (CCEB),^10^ which evaluated the socioeconomic profile of the participants’ families. CCEB has nine questions concerning the ownership of household items (color television, radio, bathroom, car, domestic worker, washing machine, VCR/DVD, refrigerator, freezer) and one question about the education level of the head of the family. Each item answered receives a score, resulting in a rating ranging from A1, higher purchasing power, to E, lower purchasing power;
- Questionnaire on the child's clinical and sociodemographic data, which was filled by the attending physician. The questionnaire addressed: length of medication use, pregnancy and obstetric data, neurological examination and neurodevelopmental assessment, other treatments, sleep patterns, and behaviors. DSM-5 diagnostic criteria for ADHD were considered in the clinical evaluation;
- *Autoquestionnaire Qualité de Vie Enfant Imagé* (AUQEI),^11^ or QoL assessment scale for children and adolescents, which seeks to evaluate the individual's perceived well-being. The scale covers family and social relationships, school activities, and health through 26 questions classified into four domains: function, family, leisure, and autonomy. Questions 6, 7, 9, 12, 14, 17, 20, 22, and 26 are not included in the 4 factors and have individual importance since they represent independent domains. The cut-off point for impaired QoL was <48.

Data were electronically digitized in a Microsoft Excel^®^ spreadsheet and checked by two people for processing and analysis. The IBM Statistical Package for the Social Sciences (SPSS), version 21.0, was used for all analyses.

We performed a descriptive analysis of the categorical variables through absolute frequency distribution and a relative analysis, in addition to numerical synthesis for the continuous variables.

A new categorization was necessary to verify the association of social behavior with ADHD criteria, economic classification, and strengths and difficulties. The CCEB was determined as A1/B1/B2 and C1/C2/D, and the MTA-SNAP-IV as normal or abnormal, without distinction between hyperactive, inattentive, or combined profiles, mainly due to the small number of observations in some categories. In the association analysis, the proportional distribution of categorical variables was initially evaluated according to the final SNAP classification (normal and abnormal), using the chi-square or Fisher's exact test. For the total SDQ score, we used Student's *t*-test to compare means, as this variable presented normal distribution. The Mann-Whitney U test was adopted for age, given the asymmetric distribution of this variable.

With respect to the choice of variables and inclusion in the multivariate model, we considered significant the associations with a significance level greater than or equal to 20% (p≤0.20). In the multivariate analysis, the significance level was set at 5%, and the odds ratio (OR) and its respective confidence interval were used to measure the magnitude of association. The adequacy of the initial and final models was evaluated by the Hosmer-Lemeshow test.

This study was authorized by the institutions where the data were collected and approved by the Research Ethics Committee (REC) of the Universidade Federal de Minas Gerais (UFMG), under ethical opinion No. 2,533,807. All participants and their guardians signed the AF and ICT, respectively.

## RESULTS

Table 1 presents sociodemographic characteristics distributed according to MTA-SNAP-IV. Among the 72 children evaluated by the MTA-SNAP-IV screening instrument, 31 (43%) had the combined ADHD profile; 15 (20.8%) had the inattentive profile; 12 (16.7%) had the hyperactive profile; and 14 (19.5%) did not meet the ADHD criteria, probably because they were on drug treatment. The inattentive profile was more frequent in children aged 9 and 10 years, and the hyperactive one showed higher incidence among 9-year-olds, while the combined profile was more heterogeneous, mostly affecting children aged 8 to 11 years. The school grades with the highest number of children were the fourth (23.6%), fifth (22.2%), and sixth (20.8%) grades.

**Table 1:** Descriptive analysis of sociodemographic variables according to results from the Multimodal Treatment Study of Children with Attention-Deficit/Hyperactivity Disorder – Swanson, Nolan, and Pelham (n=72).

Characteristics		MTA-SNAP-IV			
		Inattentive	Hyperactive	Combined	Does not meet the criteria
		n (%)	n (%)	n (%)	n (%)
Sex					
	Male	9 (60)	10 (83.3)	23 (74.2)	12 (85.7)
	Female	6 (40)	2 (16.7)	8 (25.8)	2 (14.3)
	Total	15 (100)	12 (100)	31 (100)	14 (100)
Age (years)					
	6	1 (6.7)	1 (8.3)	1 (3.2)	0 (0)
	7	0 (0)	1 (8.3)	3 (9.7)	3 (21.4)
	8	1 (6.7)	2 (16.7)	6 (19.4)	0 (0)
	9	4 (26.7)	5 (41.7)	5 (16.0)	4 (28.6)
	10	4 (26.7)	1 (8.3)	6 (19.4)	2 (14.3)
	11	2 (13.3)	2 (16.7)	6 (19.4)	4 (28.6)
	12	3 (19.9)	0 (0)	3 (9.7)	0 (0)
	13	0 (0)	0 (0)	1 (3.2)	1 (7.1)
	Total	15 (100)	12 (100)	31 (100)	14 (100)
School grade					
	1^st^	0 (0)	1 (8.3)	1 (3.2)	0 (0)
	2^nd^	0 (0)	1 (8.3)	3 (9.7)	2 (14.3)
	3^rd^	1 (6.7)	3 (25)	6 (19.4)	2 (14.3)
	4^th^	4 (26.7)	4 (33.4)	5 (16.1)	4 (28.6)
	5^th^	5 (33.3)	1 (8.3)	8 (25.8)	2 (14.3)
	6^th^	5 (33.3)	2 (16.7)	5 (16.1)	3 (21.4)
	7^th^	0 (0)	0 (0)	3 (9.7)	1 (7.1)
	Total	15 (100)	12 (100)	31 (100)	14 (100)
CCEB					
	A2	1 (6.7)	0 (0)	0 (0)	1 (7.1)
	B1	0 (0)	1 (8.3)	2 (6.7)	0 (0)
	B2	2 (13.3)	1 (8.3)	7 (22.6)	6 (42.9)
	C1	5 (33.3)	7 (58.3)	9 (30.0)	2 (14.3)
	C2	6 (60)	1 (8.3)	8 (25.8)	4 (28.6)
	D	1 (6.7)	2 (16.7)	5 (16.1)	1 (7.1)
	Total	15 (100)	12 (100)	31 (100)	14 (100)
Guardian's education level					
	Incomplete elementary school	2 (13.3)	3 (25)	9 (30)	2 (14.3)
	Complete elementary school	1 (6.7)	2 (16.7)	4 (13.3)	1 (7.1)
	Incomplete high school	1 (6.7)	1 (8.3)	2 (6.7)	1 (7.1)
	Complete high school	9 (60)	3 (25)	14 (46.7)	8 (57.3)
	Complete higher education	2 (13.3)	3 (25)	1 (3.3)	1 (7.1)
	Graduate degree	0 (0)	0 (0)	0 (0)	1 (7.1)
	Total	15 (100)	12 (100)	31 (100)	14 (100)

CCEB: Brazilian Economic Classification Criteria; MTA-SNAP-IV: Multimodal Treatment Study of Children with Attention-Deficit/Hyperactivity Disorder – Swanson, Nolan, and Pelham, version IV.

As for CCEB, only two children were classified as A2, while most belonged to classes C1 and C2 (n=42). In addition, most guardians had completed high school or higher education (n=41). However, an expressive number of guardians had not finished elementary school (n=16), as can also be seen in Table 1.

**Chart 1:**
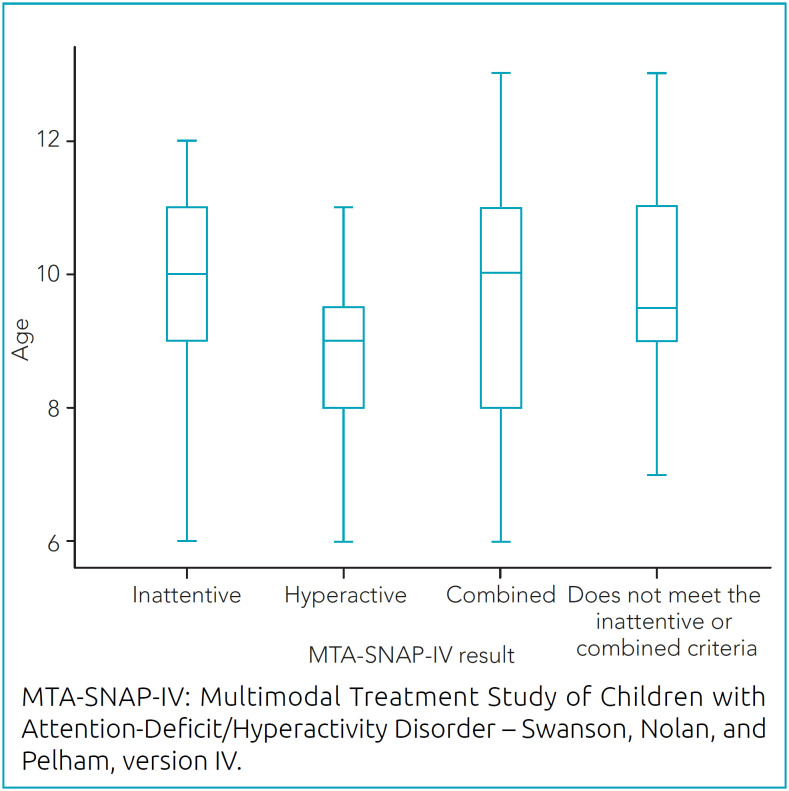
Age distribution according to results from the Multimodal Treatment Study of Children with Attention-Deficit/Hyperactivity Disorder – Swanson, Nolan, and Pelham, version IV.

The mean age was 8.8 years among children with the hyperactive profile and 9.9 among children with the inattentive profile. Children with the combined profile and those who did not meet the ADHD criteria presented the oldest ages (Chart 1).

**Chart 2:**
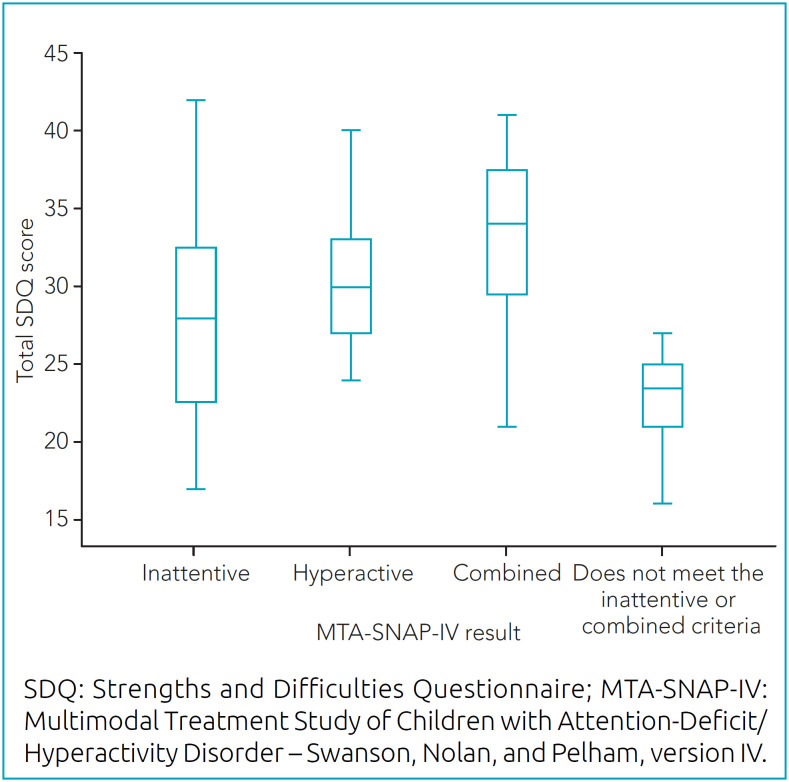
Distribution of the total score of the Strengths and Difficulties Questionnaire according to results from the Multimodal Treatment Study of Children with Attention-Deficit/Hyperactivity Disorder – Swanson, Nolan, and Pelham.

When evaluating the SDQ instrument among children with the inattentive profile, the mean score was 28.5 (result considered abnormal), ranging from 17 to 42. Among those with the hyperactive profile, the lowest score was 24 (abnormal) and, in those with the combined profile, the mean score was 32.8 (abnormal). The SDQ score distribution was more heterogeneous compared to the MTA-SNAP-IV score, with a lower mean among those who did not meet the classification criteria — 22.9 (standard deviation [SD]=3.3) —, followed by the inattentive, hyperactive, and combined types, whose averages were 28.5, 30.3, and 32.8, respectively (Chart 2).

The inferential analysis between the ADHD subtypes using the MTA-SNAP-IV and the explanatory variables required a new categorization of the variables, given the small number of observations. We found that most children were attending grades 1 to 4 (51.4%), the predominant social class was C/D (70.8%), and most parents or guardians had completed high school or higher education (59.2%). Also, most participants presented abnormal MTA-SNAP-IV (80.6%) (Table 2).

**Table 2:** Distribution of sociodemographic variables and results from the Multimodal Treatment Study of Children with Attention-Deficit/Hyperactivity Disorder – Swanson, Nolan, and Pelham (n=72).

		n	%
Current grade			
	1^st^ to 4^th^ grade	37	51.4
	5^th^ to 7^th^ grade	35	48.6
	Total	72	100
CCEB			
	A2/B1/B2	21	29.2
	C1/C2/D	51	70.8
	Total	72	100
Guardian's education level*			
	Incomplete/complete elementary school	29	40.8
	High school/higher education	42	59.2
	Total	71	100
MTA-SNAP-IV result			
	Normal	14	19.4
	Abnormal	58	80.6
	Total	72	100

* n=71; CCEB: Brazilian Economic Classification Criteria; MTA-SNAP-IV: Multimodal Treatment Study of Children with Attention-Deficit/Hyperactivity Disorder – Swanson, Nolan, and Pelham, version IV.

Table 3 shows the analysis of the association of sociodemographic variables and QoL with results from MTA-SNAP-IV. The findings revealed no statistically significant association between social class and MTA-SNAP-IV results. Regarding the children's QoL, evaluated by the AUQEI instrument, 69.4% of participants considered it good.

**Table 3:** Association between sociodemographic variables and results from the Multimodal Treatment Study of Children with Attention-Deficit/Hyperactivity Disorder – Swanson, Nolan, and Pelham (n=72).

Characteristics		MTA-SNAP-IV		
		Normal (n=14)	Abnormal (n=58)	p-value
		n (%)	n (%)	
Sex				
	Male	12 (85.7)	42 (72.4)	0.253
	Female	2 (14.3)	16 (27.6)	
School grade				
	1^st^ to 4^th^	8 (57.1)	29 (50)	0.631
	5^th^ to 7^th^	6 (42.9)	29 (50)	
Guardian's education level				
	Incomplete/complete elementary school	4 (28.6)	25 (43.9)	0.297
	High school/higher education	10 (71.4)	32 (56.1)	
CCEB				
	A2/B1/B2	7 (50)	14 (24.1)	0.060
	C1/C2/D	7 (50)	44 (75.9)	
Quality of life				
	Poor	4 (28.6)	18 (31)	0.567
	Good	10 (71.4)	40 (69)	

CCEB: Brazilian Economic Classification Criteria; MTA-SNAP-IV: Multimodal Treatment Study of Children with Attention-Deficit/Hyperactivity Disorder – Swanson, Nolan, and Pelham, version IV.

In the multivariate analysis, the only variable that remained associated with the MTA-SNAP-IV result was SDQ (Table 4). A 1-point increment in the SDQ score increased by 36.5% the likelihood of the child having an abnormal classification (OR 1.36; 95%CI 1.14–1.62; p<0.001).

**Table 4:** Multivariate analysis of selected variables and results from the Multimodal Treatment Study of Children with Attention-Deficit/Hyperactivity Disorder – Swanson, Nolan, and Pelham (n=72)#.

	Abnormal MTA-SNAP-IV result					
	Initial model			Final model		
	OR	95%CI	p-value*	OR	95%CI	p-value*
CCEB – C1/C2/D	3.31	0.73 - 15.02	0.067	−	--	−
SDQ	1.38	1.14 - 1.16	0.001	1.36	1.14 - 1.,62	<0.001

* Wald test

# reference categories: Brazilian Economic Classification Criteria (CCEB) — classes A2/B1/B2. Initial/final model adjustment (Hosmer-Lemeshow): initial model, p=0.772; final model, p=0.735; MTA-SNAP-IV: Multimodal Treatment Study of Children with Attention-Deficit/Hyperactivity Disorder – Swanson, Nolan, and Pelham, version IV; OR: odds ratio; 95%CI: 95% confidence interval; SDQ: Strengths and Difficulties Questionnaire.

## DISCUSSION

The present study detected an association between SDQ and MTA-SNAP-IV, used in the *Multimodal Treatment Study of Children with Attention-Deficit/Hyperactivity Disorder* and validated in Brazil in 2006.^12^

This research identified a predominance of male children, who represented three-quarters of the sample. Similar data were found in other investigations, which also revealed a prevalence of male children.^13^

More than half of the families evaluated belonged to social classes C and D, and about one-quarter of guardians had not finished elementary school. This information is relevant when considering the data collection scenario. The study site is in a metropolitan area in the inland of Minas Gerais, with patients from 24 cities of the metropolitan belt, a population of approximately 1 million inhabitants, and a human development index (HDI) of 0.745 — considered high;^14^ however, this index results from the 4 main industrial cities in the region, a situation not individually reflected in the other 20 cities of the belt. In addition, the literature indicates a relationship between vulnerability, ADHD, and difficult access to treatment.^15^

The analysis of predominant ADHD profiles detected a higher proportion of female participants with the inattentive profile and male ones with hyperactive and combined profiles. These data corroborate literature findings, which indicate a higher number of hyperactive boys and inattentive girls.^16^

Another finding related to the predominantly hyperactive profile was the mean age — about one year younger than children with the inattentive profile. A possible justification for this result is the fact that hyperactive and impulsive children draw the attention of caregivers and of the school staff to the need for evaluation, which would not necessarily happen with inattentive children. This finding was also identified in the literature, which revealed a delay in the diagnosis and start of treatment among inattentive female children.^17^ ^,^ ^18^

This study found that more than three-quarters of the children evaluated presented abnormal MTA-SNAP-IV, even though all of them received medical follow-up.

Concerning multivariate analysis data, the SDQ score was related to the likelihood of the child presenting abnormal MTA-SNAP-IV. In other words, a 1-point increment in the SDQ score increased by 36.5 the chance of the child having abnormal MTA-SNAP-IV. Thus, children with abnormalities were more likely to present other neuropsychiatric disorders, such as a higher level of anxiety, depression, and conduct disorders. We found no comparative research on these two instruments, but studies have revealed the importance of evaluating screening instruments, such as SNAP-IV and SDQ, in children diagnosed with ADHD to identify neuropsychiatric comorbidities associated with the disorder.^19^ ^,^ ^20^

Other studies have shown that children with ADHD presented a high number of internalizing and externalizing neuropsychiatric comorbidities, reaching up to two-thirds of individuals diagnosed with ADHD.^21^ In the present study, the number of children with a good QoL may reflect the intervention and follow-up performed.

We underline that the outpatient clinics where data were collected are specialized in pediatric neurology, which may have influenced their greater demand by guardians of children with more severe ADHD.

The limitations of this study include the use of screening instruments in a population already diagnosed and on ADHD treatment, as these patients are known to present comorbidities, which might confound data interpretation, as well as the evaluation in only two treatment centers for children with the disorder. Also, since the sample is not population-based, its representativeness decreased, despite the performance of sample calculation to identify the number of cases needed for the study.

As strong points, we highlight the relationship between SDQ and MTA-SNAP-IV and the importance of evaluating instruments that may suggest comorbidities and other neuropsychiatric disorders, in addition to the comparison with diagnoses made in referral centers for the treatment of children with ADHD.

We emphasize that this study revealed the need for interdisciplinarity and communication between neurology and other specialties, such as psychiatry, psychology, and psychopedagogy, in the treatment of children with ADHD.

Thus, this study confirmed the association between clinical characteristics of children with ADHD and sociodemographic aspects. Among them, we can mention: belonging to social class C and the inattentive profile in children aged 8/9 years. As to QoL, we found no relationship between the data assessed and ADHD, since most participants presented good QoL. The use of screening instruments to identify comorbid neuropsychiatric disorders and their relationship with the ADHD profile revealed that children with abnormal MTA-SNAP-IV were more likely to also have abnormal SDQ.

Further research is needed on practical approaches to detect neuropsychiatric disorders in children with ADHD and the use of screening instruments in order to increase diagnostic validity, favoring a collaborative mental health model.
